# A Synthetic Manassantin A Derivative Inhibits Hypoxia-Inducible Factor 1 and Tumor Growth

**DOI:** 10.1371/journal.pone.0099584

**Published:** 2014-06-12

**Authors:** Liwei Lang, Xiaoyu Liu, Yan Li, Qing Zhou, Ping Xie, Chunhong Yan, Xiaoguang Chen

**Affiliations:** 1 Department of Pharmacology, State Key Laboratory of Bioactive Substances and Functions of Natural Medicines, Institute of Materia Medica, Chinese Academy of Medical Sciences and Peking Union Medical College, Beijing, P.R. China; 2 Department of Synthetic Medicinal Chemistry, State Key Laboratory of Bioactive Substances and Functions of Natural Medicines, Institute of Materia Medica, Chinese Academy of Medical Sciences and Peking Union Medical College, Beijing, P.R. China; 3 GRU Cancer Center, Department of Biochemistry and Molecular Biology, Medical College of Georgia, Georgia Regents University, Augusta, Georgia, United States of America; University of Dundee, United Kingdom

## Abstract

The dineolignan manassantin A from *Saururaceae* was recently identified as a hypoxia-inducible factor 1 (HIF-1) inhibitor, but its *in-vivo* anti-tumor effect has not been explored. We synthesized a series of manassantin A derivatives, and found that replacing the central tetrahydrofuran moiety with a cyclopentane ring yielded a compound (LXY6006) with increased HIF-1-inhibitory activity yet decreased stereochemically complexity amenable to a simplified synthesis scheme. LXY6006 inhibited HIF-1α nuclear accumulation induced by hypoxia, and inhibited cancer cell growth as a consequence of G2/M arrest. Oral administration of LXY6006 significantly inhibited growth of breast, lung, and pancreatic tumors implanted in nude mice. These results indicate that LXY6006 represents a novel class of agents targeting a broad range of human cancers.

## Introduction

Solid tumors are often subjected to hypoxia and need to adapt to the hypoxic environment to sustain their rapid growth. As a transcription factor, hypoxia-inducible factor (HIF) is the master regulator of the cellular hypoxia response [1]. HIF is a heterodimer composed of a hypoxia-regulated subunit (HIF-α) and a constitutively expressed subunit (HIF-1β). Among three HIF-α isoforms, HIF-1α is the most critical regulator of hypoxia responses in solid tumors, and its activity is indispensable for tumors to adapt to hypoxia conditions and recover from damages caused by hypoxic insult [2]. While HIF-1β is constitutively expressed in the nucleus, HIF-1α remains at a low level through proteasome-dependent mechanisms under normoxia [3]. Upon hypoxia, HIF-1α is quickly stabilized and translocated to the nucleus, where it forms a heterodimer with HIF-1β and subsequently binds to the hypoxia responsive element (HRE) (5′-RCGTG-3′), resulting in transactivation of more than 200 genes required for the cell to adapt to hypoxic conditions [4]. Since many of the HIF-1 target genes can promote cell survival under hypoxic conditions, it is not surprising that HIF-1α is often overexpressed in various cancers, including breast, lung, pancreatic and renal cancer. Therefore, inhibition of HIF-1α activity represents an attractive strategy for cancer treatment. Indeed, small molecules targeting HIF-1α transcription, translation, and stabilization have been developed, and some of them (e.g., PX-478, Topetican and BAY87-2243) have entered clinical trials for treating cancer patients [5].

Manassantin A is a dineolignan isolated from the herb *Saururus cernuus* (Saururaceae) used for treating edema, gonorrhea and jaundice in Asia. At micromolar concentrations, manassantin A was shown to inhibit NF-κB activation and nitric oxide production in macrophages, block MAPK activation in mast cells, and inhibit transcription of a wide range of genes in various cell types [6]–[8]. Manassantin A was recently identified as a HIF-1 inhibitor with IC_50_ values ranging from 1 to 10 nM through a cell- and reporter-based screening [9], [10]. This compound inhibits HIF-1 activity by blocking hypoxia-induced nuclear HIF-1α accumulation without altering HIF-1α transcription [9]. Moreover, manassantin A inhibits growth of cultured cancer cells under normoxia conditions through unknown mechanisms [11]. Although its anti-tumor activity has not yet been determined in animal models, manassantin A may serve as an ideal lead for further drug development.

**Figure 1:**
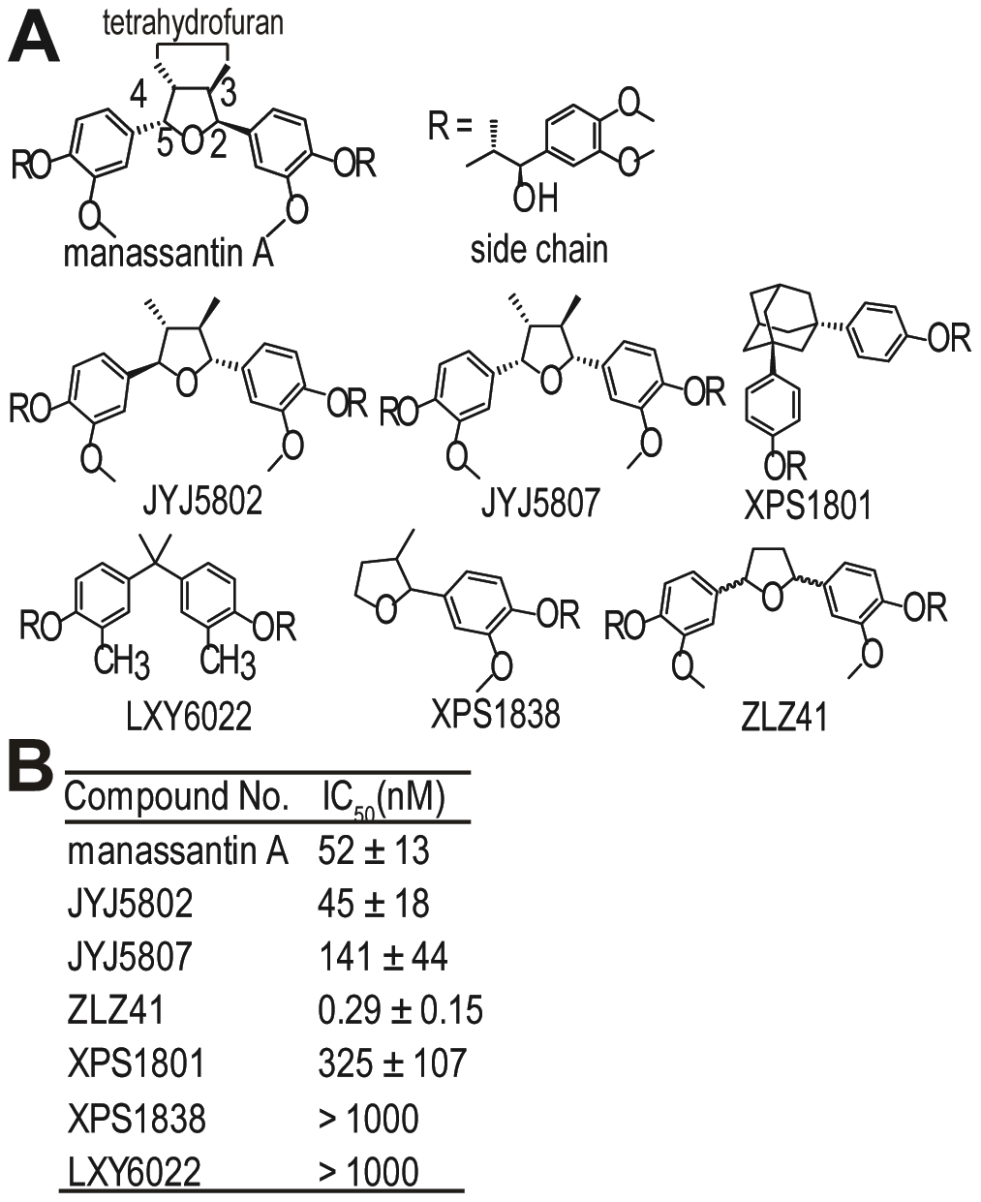
The HIF-1-inhibitory activity of manassantin A derivatives. (A) Structures of manassantin A and synthesized derivatives. (B) HIF-1 inhibitory activity of manassantin A derivatives measured by a HIF-1 reporter assays.

Manassantin A has eight chiral centers and a 2,3-cis-3,4-trans-4,5-cis-configuration at the central tetrahydrofuran core (Fig 1A). As the tetrahydrofuran moiety is chemically and stereochemically complex, synthesis of manassantin A is difficult. In an attempt to optimize the structure of manassantin A, we developed a compound LXY6006 by replacing the tetrahydrofuran moiety with a cyclopentane ring. We found that this novel, simplified compound inhibited HIF-1 activity more potently than manassantin A while it also exhibited anti-cancer activities independent of HIF-1 inhibition. Importantly, oral adminstration of LXY6006 inhibited xenograft tumor growth without altering animal weights. Therefore, LXY6006 has a potential to be further developed into a therapeutic agent for treating various solid tumors.

## Materials and Methods

### Chemicals

**Figure 2:**
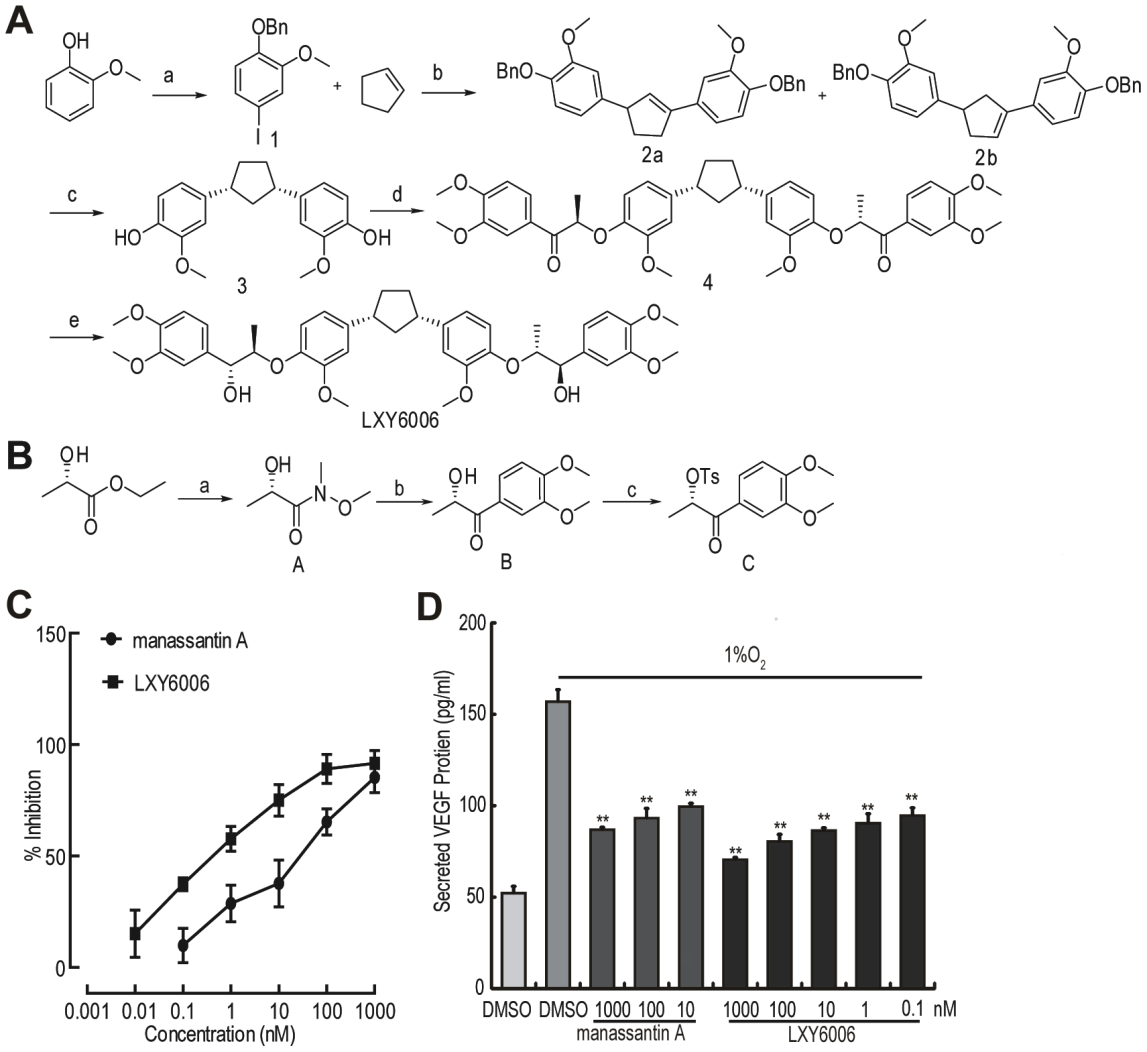
LXY6006 is a novel HIF-1 inhibitor derived from manassantin A. (A) The scheme for LXY6006 synthesis. Reagents and conditions: (a) BnBr, K_2_CO_3_, I_2_, NaOH; (b) Pd (OAc)2, n-Bu4NCl, KOAc, DMF, 80°C, 4 hr, 73.2%; (c) H2, Pd-C, 25°C, 2 hr, 87.5%; (d) PS-BEMP, CH3CN, 25°C, 10 hr, 97.5%; (e) PS-brohydride, CH3OH, 25°C, 28 hr, 99.5%. (B) The scheme for LXY6006 side chain synthesis. Reagents and conditions: (a) Isopropylmagnesium chloride, N,O-dimethyl Hydroxylamine hydrochloride, THF, −20°C; (b) n-BuLi, 3,4-dimethylbromobenzene,THF, −78°C; (c) Ts_2_O, Pyridine, 0°C. (C) LXY6006 inhibited HIF-1 activity more potently than manassantin A. T47D cells transfected with the HIF-1 reporter were treated with various concentrations of manassantin A and LXY6007 for 16 hr in 1%O_2_ and subjected to dual luciferase activity assays. Data are shown as means ±SD from one representative experiment performed in triplicate. (D) LXY6006 inhibits VEGF protein expression in T47D cells. T47D cells were treated with LXY6006 and manassantin A, and then incubated in a hypoxia chamber (1%O_2_) for 16 hr. The VEGF amount in conditioned medium was determined by ELISA. Data are shown as means ±SD from one representative experiment performed in triplicate. **p<0.01(Student t-test), compared to the DMSO (1%O_2_) group.

Chemicals used for synthesis were purchased from Alfa Aesar Co. Ltd. (MA, USA), Acros Organics (Geel, Belgium), or Sigma-Aldrich (MO, USA). LXY6006 was synthesized as described in Fig 2A and 2B. In brief, aromatic iodide (**1**) was first synthesized, and 1.3-diacrylcyclopentene (**2a** and **2b**) was generated through the Double Heck arylation reaction under mild solid-liquid Phase Transfer Catalysis (PTC) conditions reported by Jeffery [12], Larock [13] and Prashad [14]. The Bn groups were then removed through a Pd-C catalyzed reaction at room temperature, resulting in Compound **3** (Fig 2A). The side chain bis-ketone (**4**) was obtained through amidation of L-ethyl lactate followed by a reaction with the Grignard reagent and tosylation of α-hydroxy ketone (**6**) using toluenesulfonic anhydride and pyridine (Fig 2B). Finally, LXY6006 was synthesized through a BEMP-mediated SN2 reaction of **3** followed by stereocontrolled reduction using polymer-supported BH_4_ (Fig 2A). The purity of LXY6006 used in this study was 96.5% as measured by HPLC. For treating cultured cells, tested chemicals were dissolved in DMSO (Sigma). For animal experiments, 1 mg/ml or 3 mg/ml taxol (paclitaxel injection, Union Pharmaceutical Factory, Beijing, China), or 15 mg/ml Gemcitabine (Lily, France) was prepared freshly with sterile saline. LXY6006 was prepared in 0.5% carboxy methylated cellulose as a 3 or 6 mg/ml stock solution and stored at −20°C before use.

### Cell culture and viability assay

MX-1 and MX-1/Taxol cells were kindly provided by Dr. Yongkui Jing (Mount Sinai School of Medicine, NY, USA) [15]. Other cell lines used in this study were purchased from ATCC or the Cell Culture Center at Institute of Basic Medical Sciences, Chinese Academy of Medical Sciences. Cells were maintained in DMEM, DMEM/F12, or RPMI-1640 supplemented with 10% fetal bovine serum (Gibco, USA) in a humidified incubator containing 5% CO_2_ at 37°C as required. Cell viability after treatments with tested compound for 5 days were measured in 96-well plates by MTT assays as described [16]. IC_50_ values (50% inhibitory concentration) were calculated using the software GraphPad Prism 5.0.

### HIF-1 reporter activity assays and ELISA

T47D cells in 96-well plates were co-transfected with 0.01 µg pRL-CMV (Promega, USA) and 0.2 µg pGL2-TK-HRE carrying a firefly luciferase gene driven by 3 tandem repeats of HRE sequences [17] by lipofectamine 2000 (Invitrogen, USA) following manufacturer's instruction. 24 hr later, cells were treated with test compounds for 30 min, moved to a hypoxia chamber (5%CO_2_, 1%O_2_, 94%N_2_), and incubated for 16 hr for dual luciferase activity assays (Promega). For ELISA, T47D cells in 96-well plates were treated as above, and condition media were transferred to a new plate 16 hr later. VEGF amounts were determined following the manufacturer's instruction (R&D Systems, USA).

### Western blotting

Exponentially growing cells were treated with chemicals before exposed to normoxia (21%O_2_), 1,10-phenanthroline(10 µM), or hypoxia (1%O_2_) at 37°C. Nuclear extracts were prepared using the Nuclear and Cytoplasmic Extraction Reagents (CWBio Co., Beijing, China). Cells were lysed using RIPA buffer containing 50 mM Tris-HCl, pH7.4, 150 mM NaCl, 1% Triton X-100, 0.5% deoxycholate, 0.1% SDS, and protease inhibitor cocktails (Amresco, USA). Equal amounts of proteins were resolved by SDS-PAGE gels and transferred to PVDF membranes (Millipore, USA). Membranes were blocked with 5% non-fat dry milk followed by incubation with primary antibodies and HRP-conjugated secondary antibodies for protein visualization. The HIF-1α antibody and the HIF-1β antibody were purchased from R&D Systems (USA) and Santa Cruz (USA), respectively. Other antibodies were purchased from CST (MA, USA).

### Immunofluorescence staining

T47D cells on glass coverslips were treated and exposed to normoxic (21%O_2_) or hypoxic (1%O_2_) conditions at 37°C for 8 hr as described above. After fixed with 4% formaldehyde for 30 min, cells were permeabilized with 0.5% Triton X-100 for 20 min and then blocked with normal goat serum for 1 hr. Cells were then incubated with the HIF-1α (1∶50) antibody at 4°C overnight, followed by incubation with the Dylight 594-conjugated goat anti-mouse antibody (1∶400, ZhongShan Golden Bridge, Beijing, China) at room temperature for 40 min. Cells were counter-stained by DAPI (1 µg/ml), and observed under a fluorescence microscope (Kodak America, Inc.).

### Flow cytometry

T47D cells treated with manassantin A or LXY6006 for 4 or 5 days were harvested with 0.2% trypsin, washed in PBS, and then fixed in 70% ethanol at −20°C overnight. After washed with PBS, cells were stained with 50 µg/ml propidium iodide (PI) in the presence of RNase (30 U/ml) at 37°C for 30 min, and subjected to flow cytometry analysis. The percentage of apoptotic cells was determined based on sub-G1/G0 populations.

### Xenograft tumor growth

The animal protocol was approved by the Experimental Animal Management and Welfare Committee at the Institute of Materia Medica, Peking Union Medical College. Athymic nude mice (BALB/c-nu/nu females, 6–8 weeks old) were purchased from Vital River Laboratory Animal Technology Co., Ltd. (Beijing, China) and housed in controlled environment at 25°C on a 12-h light/dark cycle. To establish MX-1, H-460, MIA Paca-2, and MX-1/Taxol xenograft, 1×10^7^ cells were injected subcutaneously into flanks of nude mice and grew for several weeks. The tumors were then chopped into 3×3×3 mm^3^ pieces and implanted subcutaneously at flanks of mice with a gauge trocar. When tumors reached to 100–200 mm^3^, mice were randomized into different groups (6–7 mice per group), and then treated with tested compounds. Taxol (10 mg/kg, or 30 mg/kg) and Gemcitabine (150 mg/kg) were injected intraperitoneally twice weekly. LXY6006 was given to mice by intragastric administration 6 days per week. Tumor volume, relative tumor volume were calculated as described before [18].

### Statistical analysis

Data are expressed as means ± standard deviation. Statistical analysis was performed with the Mann-Whitney U test or Student t-test using SPSS 15.0.

## Results

### A novel manassantin A derivative LXY6006 inhibits the HIF-1 transcription activity

The natural product manassantin A is a strong HIF-1 inhibitor, but its total synthesis is cumbersome due to structure complexity. We sought to optimize the structure of manassantin A and explore anti-cancer effects of its derivatives. Using a published method [19], we synthesized manassantin A, its stereoisomers and a series of derivatives (Fig 1A), and determined their HIF-1 inhibitory activities through a cell-based HIF-1 reporter assay [17]. While the change of the tetrahydrofuran configuration (JYJ5802 and JYJ5807) only slightly altered the HIF-1 inhibitory activity (Fig 1A and 1B), the central tetrahydrofuran moiety appears to be important for HIF-1 inhibition as its replacement with other ring moieties or an aliphatic core (XPS1801 and LXY6022) dramatically decreased the HIF-1 inhibitory activity (Fig 1A and 1B). Two side chains were also indispensable as removal of one side chain resulted in an inactive compound (XPS1838) (Fig 1A and 1B). On the contrary, removal of the two methyl groups from the tetrahydrofuran core (ZLZ41) increased the activity by more than 150 folds (Fig 1A and 1B). Based on these results, we synthesized a compound (cis-1,3-bis(3’’-methoxy-4’’-phenyl-7’’’R-8’’’R-3’’’,4’’’-dimethoxy-7’’’-hydroxy-8’’’-methylphenylethane)ether), referred to as LXY6006 thereafter (Fig 2A), by replacing the tetrahydrofuran core with a 1,3-cis-cyclopentane ring (Fig 1A and 2A). The synthesis of LXY6006 (Fig 2A and 2B) was much easier than that of manassantin A due to the fact that the cyclic or acyclic substituents are readily accessible. Importantly, this derivative inhibited HIF-1 activity more efficiently than manassantin A (IC50 = 0.35±0.11 nM vs. 52±9 nM for manassantin A) (Fig 2C). Consistent with its HIF-1 inhibitory activity, hypoxia-induced expression of vascular endothelial growth factor (VEGF), a known HIF-1 target gene [20], was significantly down-regulated by LXY6006 (Fig 2D).

### LXY6006 blocks hypoxia-induced HIF-1α nuclear accumulation

**Figure 3:**
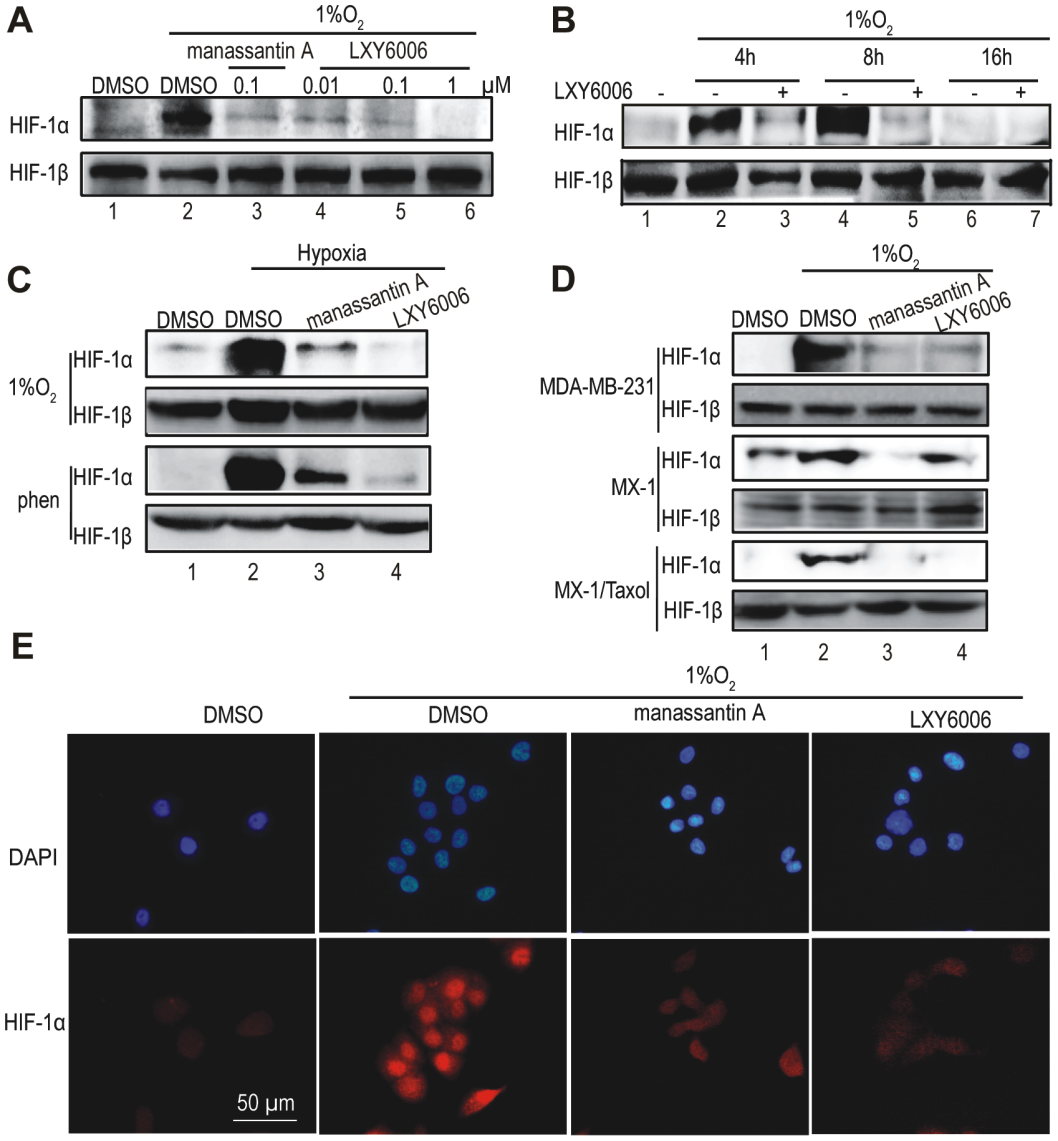
LXY6006 inhibits HIF-1α nuclear accumulation. (A) Nuclear extracts from T47D cells were subjected to Western blotting for HIF-1α and HIF-1β expression. HIF-1β was constitutively expressed and thus used as a loading control. (B) T47D cells pre-treated with or without 0.1 µM of LXY6006 were incubated in hypoxia conditions for different time, and then subjected to Western blotting. (C) T47D cells pretreated with or without 0.1 µM of manassantin A or LXY6006 were exposed to hypoxia (1%O_2_, 8 hr) or 1,10-phenanthroline (phen) (10 µM, 8 hr), and then lysed for detection of nuclear HIF-1α expression as in (A). (D) MDA-MB-231, MX-1 and MX-1/Taxol cells were treated with or without 0.1 µM of manassantin A or LXY6006 and lysed for HIF-1α expression as above. (E) T47D cells treated with or without 0.1 µM of LXY6006 or manassantin A were exporesed to hypoxia for 8 hr, and then fixed for immunofluorescence straining for HIF-1α expression.

Manassantin A inhibited HIF-1 activity by blocking hypoxia-induced HIF-1α accumulation in the nucleus. To test whether LXY6006 exerts a similar effect, we carried out Western blotting using nuclear extracts from T47D cells treated with LXY6006. While culturing cells in a physiological hypoxia condition (1% O_2_) significantly increased the nuclear HIF-1α level but did not alter HIF-1β level (Fig 3A, lane 2 vs. lane 1), both manassantin A and LXY6006 indeed blocked HIF-1α accumulation induced by hypoxia (Fig 3A). Hypoxia rapidly induced HIF-1α accumulation within 4-hr, and LXY6006 blocked such accumulation from the beginning (Fig 3B). Moreover, we found that both manassantin A and LXY6006 effectively decreased the nuclear HIF-1α amount induced by 1,10-phenanthroline (Fig 3C). In addition to T47D cells, LXY6006 also blocked hypoxia-induced HIF-1α accumulation in other tested breast cancer cells (Fig 3D). To corroborate these observations, we examined HIF-1 α subcellular localization by immunocytostaining. While HIF-1α was indeed accumulated in the nuclei of T47D cells, LXY6006 as well as manassantin A prevented HIF-1α from nuclear accumulation (Fig 3E). Therefore, as a novel manassantin A derivative, LXY6006 inhibited HIF-1 activity through blocking hypoxia-induced HIF-1α nuclear accumulation.

### LXY6006 selectively inhibits growth of a subset of cancer cell lines

In addition to inhibiting HIF-1, manassantin A appears to affect expression of a wide range of genes and was recently shown to inhibit cancer cell growth under normoxia conditions. To evaluate anti-cancer activity of LXY6006, we treated 19 different cancer cell lines with LXY6006, manassantin A or Taxol for MTT assays. LXY6006 as well as manassantin A selectively inhibited growth of a subset of lung, breast and pancreatic cancer cell lines, including two of the three Taxol-resistant cells (Table 1). Interestingly, LXY6006 appears to inhibit cell growth more effectively than manassantin A, with IC_50_ values which were often 5- to 100-fold smaller than that of manassantin A (Table 1). These results suggest that LXY6006 might also inhibit cell growth through HIF-1-independent mechanisms.

**Table 1:** Selective inhibition of growth of cultured cancer cells by LXY6006.

Cell line	Origin	Growth inhibition	(IC_50_nM)
		manassantin A	LXY6006	Taxol
A375	Melanoma	>1000	>1000	22.5±12.1
U251	Glioblastoma	>1000	>1000	9.5±5.4
HepG2	Hepatocellular carcinoma	>1000	>1000	23.1±8.2
ACHN	Renal cell adenocarcinoma	>1000	>1000	42.3±14.7
BGC-823	Stomach adenocarcinoma	>1000	>1000	7.2±2.4
HT-29	Colon adenocarcinoma	>1000	>1000	6.4±3.3
HCT-8	Colon adenocarcinoma	>1000	>1000	250.4±89.2
NCI-H460	Lung adenocarcinoma	>1000	>1000	68.5±26.1
A549	Lung adenocarcinoma	230.2±87.5	4.5±1.3	24.6±9.2
A549/Taxol	Lung adenocarcinoma	150.3±35.1	1.3±1.6	>1000
MCF-7	Breast adenocarcinoma	>1000	>1000	4.4±1.6
T47D	Breast adenocarcinoma	840.2±235.4	63.3±10.5	5.2±3.7
MD-MBA-231	Breast adenocarcinoma	290.7±69.4	52.6±19.3	3.2±2.8
MX-1	Breast adenocarcinoma	21.1±7.5	63.9±31.4	52.4±12.7
MX-1/Taxol	Breast adenocarcinoma	74.6±15.2	85.1±29.3	>1000
SW-1990	Pancreatic adenocarcinoma	>1000	249.7±60.1	29.5±15.2
Capan-2	Pancreatic adenocarcinoma	120.7±34.5	19.7±7.3	11.0±6.3
PANC-1	Pancreatic adenocarcinoma	340.4±120.3	78.8±31.6	2.9±1.2
MIA PaCa-2	Pancreatic adenocarcinoma	1.6±1.0	1.8±0.7	1.9±1.3

### LXY6006 induces cell cycle arrest but not apoptosis

**Figure 4:**
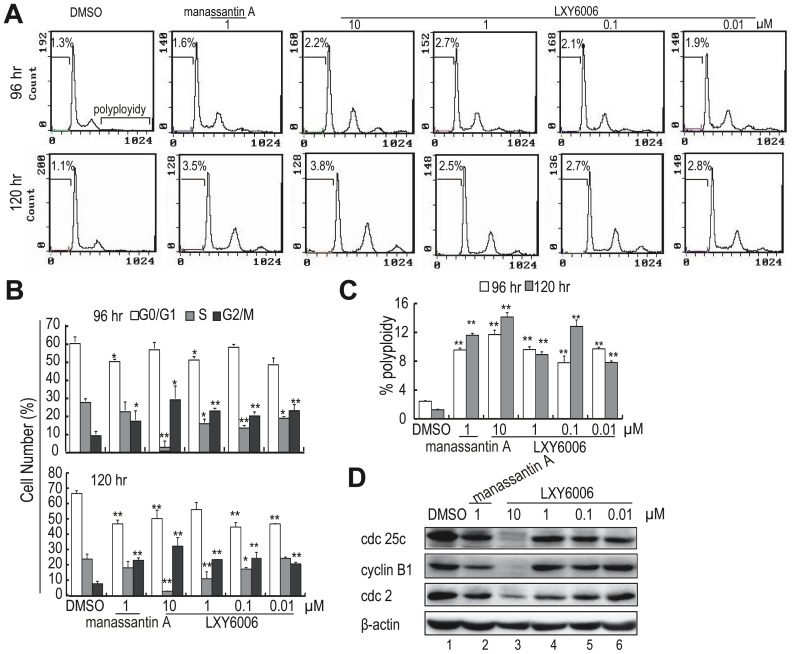
LXY6006 induces cell cycle arrest but not apoptosis. (A, B, C) T47D Cells were treated with various concentrations of LXY6006 or 1 µM of manassantin A for 4 days and 5 days, and subjected to flow cytometry for cell cycle analysis. The percentages of cells in each cell cycle phase (G1, S, and G2/M) were determined by flow cytometry. The percentage of apoptotic cells was measured by the accumulation of cells with a sub-G1 DNA content. The percentages of polyploid cells were showed in (C). *p<0.05, **p<0.01 vs. DMSO (n = 3). (D) T47D cells were treated with manssantin A or LXY6006 for 5 days for Western blotting to detect cdc25c, cyclin B1 and cdc2 expression.

Although manassantin A and LXY6006 significantly decreased viable cell numbers in our MTT assays, we noticed that these treatments did not result in apparent cell death, suggesting that manassantin A and its derivative likely induced cell cycle arrest. To test this, we carried out cell cycle analysis. Indeed, treating T47D cells with 10 nM of LXY6006 was sufficient to induce cell cycle arrest at G2/M phase (Fig 4A, B), while LXY6006 only marginally increased the number of apoptotic cells (subG1/G0, Fig 4A). In addition, LXY6006 treatments resulted in an increase in the number of polyploid cells (Figure 4A, C). To explore the mechanisms underlying these effects, we determined expression of several G2/M checkpoint genes (*i.e.*, Cyclin B1, cdc2 and cdc25c) by Western blotting. LXY6006 and manassantin A significantly down-regulated cdc25c, cycle B1 and cdc2 expression (Fig 4D), consistent with their activities in inducing G2/M cell cycle arrest. Therefore, LXY6006 likely inhibited cancer cell growth by inducing cell cycle arrest.

### LXY6006 inhibits tumor xenograft growth in mice

**Figure 5:**
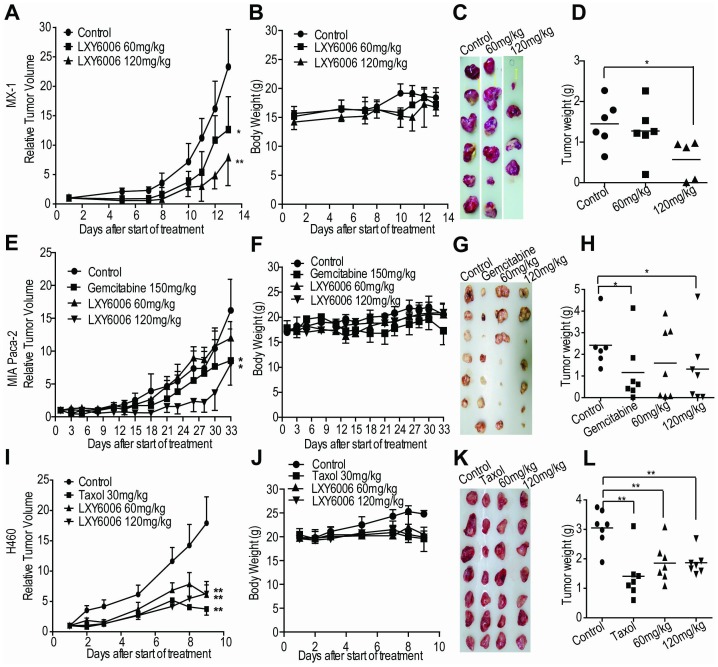
LXY6006 inhibits xenograft tumor growth. Nude mice implanted with MX-1, MIA Paca-2 or H460 xenografts, were administrated o.p. with LXY6006, or injected i.p. with Gemcitibine or Taxol as indicated. Tumor sizes were measured and used to calculate relative tumor volume (A, E, I). Body weights were monitored (B, F, J). At the end of experiments, tumors were also dissected out, and photographed (C, G, K) and weighed (D, H, L). *p<0.05, **p<0.01 vs control.

To evaluate the anti-cancer activity of LXY6006 *in vivo*, we implanted human breast (MX-1), pancreatic (MIA Paca-2) and lung (H460) tumors in nude mice, and then treated nude mice carrying established tumors with 60 and 120 mg/kg of LXY6006 (6 days per week) *via* oral administration. As positive controls, Taxol or Gemcitabine was also given to mice carrying H460 and MIA Paca-2 tumors. 120 mg/kg of LXY6006 significantly inhibited growth of these tumor xenografts (Fig 5). Not only tumor volumes (Fig 5A, 5E, and 5I) but also tumor weights (Fig 5C, 5D, 5G, 5H, 5K, and 5L) at the time of mouse sacrifice were significantly decreased by LXY6006 treatments. LXY6006 decreased the relative tumor volume to 34%, 49%, and 40% of the control for MX-1, MIA PaCa-2 and H460, respectively (Fig 5A, 5E, and 5I). Similarly, the tumor weight was reduced to 39%, 63%, and 47%, respectively (Fig 5D, 5H, and 5L). 60 mg/kg of LXY6006 also inhibited MIA Paca-2 and H460 tumor growth, but only modestly decreased the MX-1 tumor volumes. Importantly, LXY6006 only minimally affected mouse body weights during the experiments (Fig 5B, 5F and 5J), suggesting that LXY6006 was low toxic to the animals. These results indicate the potential of LXY6006 in treating breast, lung and pancreatic cancers.

### LXY6006 effectively inhibits Taxol-resistant tumor growth *in vivo*

**Figure 6:**
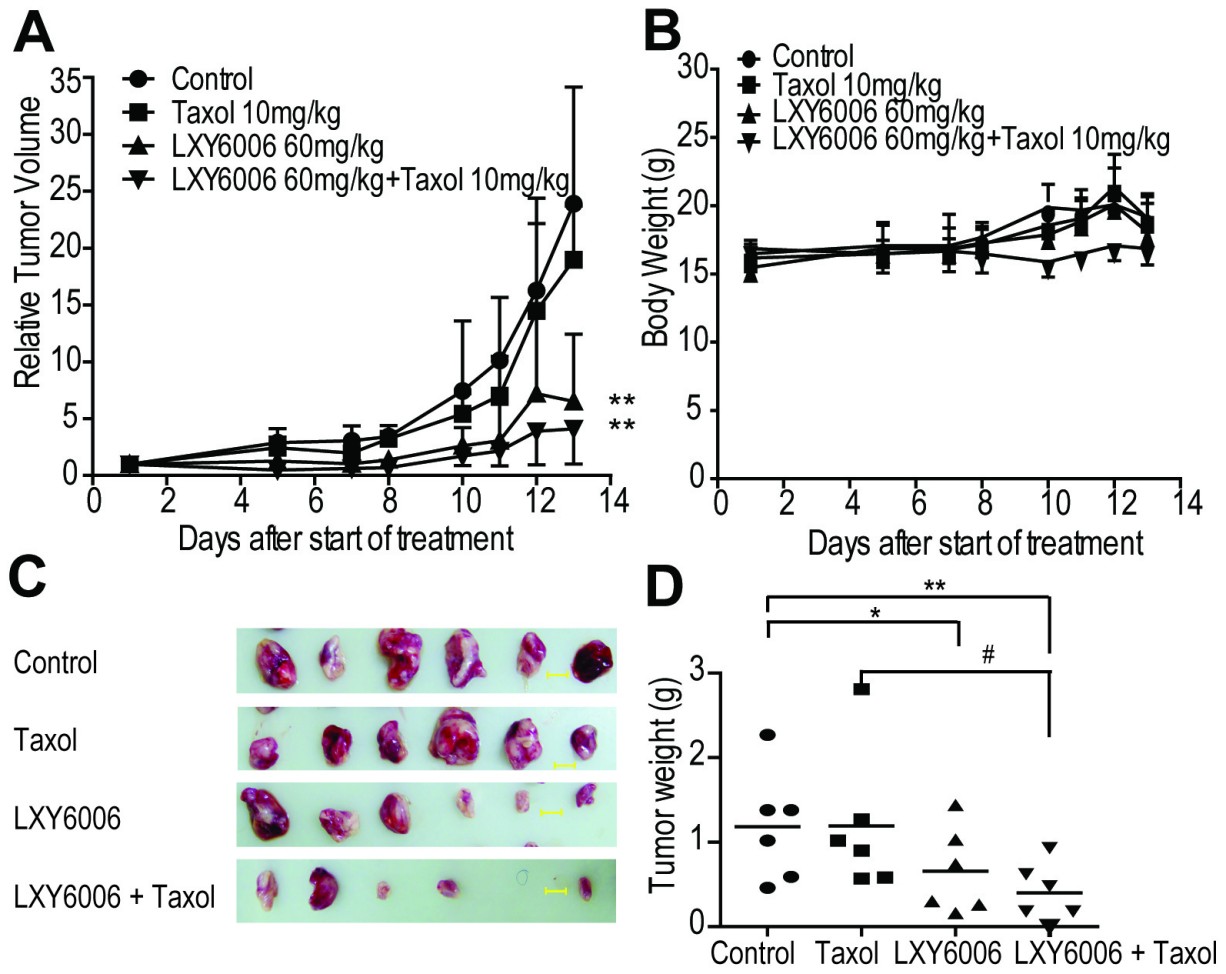
LXY6006 inhibits the growth of Taxol-resistant breast tumors. Nude mice implanted with MX-1/Taxol xenografts were treated with 60 mg/kg LXY6006, 10 mg/kg Taxol, or a combination of LXY6006 and Taxol. Tumor sizes were measured and used to calculate relative tumor volume (A). Body weights were shown in (B). Tumor were also photographed (C) and weighed (D). *p<0.05, **p<0.01 vs control; #*p*<0.05 vs LXY6006 or Taxol.

Taxol is used for breast cancer treatment, but often fails due to acquired resistance. Since our MTT results showed that LXY6006 could effectively inhibit growth of taxol resistant cancer cells (A549/Taxol, MX-1/Taxol) (Table 1), we tested if LXY6006 could be used to treat Taxol-resistant breast cancer. We thus established Taxol-resistant MX-1/Taxol tumors in nude mice, and treated the mice with 60 mg/kg of LXY6006, or Taxol. While 10 mg/kg of Taxol had a little effect on tumor growth as expected, LXY6006 significantly decreased the relative tumor volume (Fig 6A) and weight (Fig 6C and 6D) in the presence or absence of Taxol. Again, we did not observe apparent weight loss in mice (Fig 6B). Therefore, LXY6006 represents an agent alterative to Taxol for breast cancer treatment.

## Discussion

Manassantin A is an active component of the medicinal herb *Saururaceae*, and has multiple biological activities including the neuroleptic and the anti-inflammatory activity [21]. Although this natural product was identified as a potent HIF-1 inhibitor earlier [9], [10], its anti-cancer activity has not yet been tested *in vivo*. Here we showed that a manassantin A derivative LXY6006 is a more potent HIF-1 inhibitor. LXY6006 inhibited not only growth of cultured cancer cells, but also tumor xenograft growth in animals. Our results thus indicate that manassantin A and its derivatives, e.g., LXY6006, could be further developed into a novel class of anti-cancer agents targeting HIF-1-mediated pro-survival pathways.

Like manassantin A, LXY6006 inhibited HIF-1 activity through blocking hypoxia-induced accumulation of HIF-1α. HIF-1α is accumulated under hypoxia conditions through different mechanisms [22]. Although the detailed mechanism underlying this effect remains elusive, it was hypothesized that manassantin A affects expression of genes regulatory for HIF-1α accumulation. Indeed, manassantin A has been reported to affect expression of a variety of genes (*e.g.*, ICAM-1, MITF), and is also known to suppress the NF-κB transcriptional activity [23], [24]. Interestingly, manassantin A was also reported to inhibit activation of multiple signaling pathways such as pathways mediated by ERK, p38, JNK and STAT3 [25], [26]. However, manssantin A often exhibited these effects at micromolar levels. While whether these effects indeed account for mansaantin A-mediated HIF-1 inhibition remains an interesting question to be answered, the fact that LXY6006 is a more potent HIF-1 inhibitor suggests that LXY6006 could be a useful tool for dissecting the mechanism underlying the anti-cancer activity of manssantin A-derived agents.

In addition to HIF-1 inhibition, we found that LXY6006 also effectively inhibited growth of a subset of cancer cells in culture. LXY6006 exerted such anti-cancer effects through inducing cell cycle arrest rather than apoptosis. LXY6006 appeared to induce cell cycle arrest at the G2/M phase, consistent with the observations that this compound inhibited expression of G2/M checkpoint genes including cdc2, cycle B1 and cdc25c. Out of the tested G2/M checkpoint genes, cdc2 was likely the major target of LXY6006. Indeed, 10 nM of LXY6006 was sufficient to decrease the cdc2 expression level by nearly 50%. Given that manassantin A-based compounds often suppress kinase-mediated pathways at micromolar levels, the inhibition of cdc2 expression at nanomolar concentrations suggests that LXY6006 might directly bind the cdc2 promoter and inhibit cdc2 expression. It is important to note that LXY6006 inhibited T47D cell growth under a hypoxia (1%O_2_) condition at the same efficacy as it did under normoxia (21%O_2_) conditions (data not shown). Therefore, although HIF-1 has been shown to regulate cell cycle arrest, the inhibition of cdc2 expression by LXY6006 was unlikely a mere consequence of HIF-1 inhibition. Nevertheless, our results that LXY6006 selectively inhibited growth of a subset of cancer cells and that it is a potent HIF-1 inhibitor suggest that LXY6006 could be used to treat rapidly-growing tumors.

Indeed, we found that LXY6006 significantly inhibited growth of breast, lung and pancreatic tumors in nude mice. To our knowledge, our results provide the first evidence supporting that manassantin A and its derivative can be used to treat cancer *in vivo*. Interestingly, while LXY6006 did not inhibited growth of cultured H460 lung cancer cells, it significantly inhibited H460 xenograft growth in animals. Moreover, LXY6006 appeared to inhibit the growth of a Taxol-resistant breast tumor. While it would be interesting to investigate how LXY6006 might act in concert with Taxol to inhibit tumor growth, our results suggest that LXY6006 could also be used to treat cancer patients in combination with chemotherapeutic agents.
